# Supplementary material for: A Collagen Triple Helix without the Superhelical Twist
Source: ACS Cent Sci. 2025 Feb 4;11(2):331–45. doi: 10.1021/acscentsci.5c00018 (PMC11869133; doi:10.1021/acscentsci.5c00018)
Supplement: Supplementary file 1 — oc5c00018_si_001.pdf [file oc5c00018_si_001.pdf]

A Collagen Triple Helix without the Super Helical Twist

Mark A. B. Kreutzberger[1,†], Le Tracy Yu[2,†], Thi H. Bui[2], Maria C. Hancu[2], Michael D. Purdy[3], Tomasz Osinski[4], Peter Kasson[5], Edward H. Egelman[1*], Jeffrey D. Hartgerink[2,6*]

[1] Department of Biochemistry and Molecular Genetics, University of Virginia School of Medicine, Charlottesville, VA, 22903, USA
[2] Department of Chemistry, Rice University, 6100 Main Street, Houston, TX, 77005, USA
[3] Molecular Electron Microscopy Core, University of Virginia School of Medicine, Charlottesville, VA, 22903, USA
[4]Center for Advanced Research Computing, University of Southern California, Los Angeles, CA 90089
[5]Departments of Chemistry & Biochemistry and Biomedical Engineering, Georgia Institute of Technology, Atlanta, GA 30332
[6] Department of Bioengineering, Rice University, 6100 Main Street, Houston, TX, 77005, USA
[†] These two authors contributed equally to this work, listed alphabetically.
* To whom correspondence should be addressed: egelman@virginia.edu, jdh@rice.edu

Supplementary Information

Table S1. Sequences of the Collagen-like Peptide Investigated in this Study.

Peptide	Sequences
A	EDLCRAPDGKKGEAGROGRRGROGLKGEQGEOGAOGIR
B	**Q**LSCTGPOAIOGIOGIOGTOGPDGQOGTOGIKGEKGLO
C	NTGCYGIOGMOGLOGAOGKDGYDGLOGPKGEPGIO
A-ext.	EDLCRAPDG**KKGEAGROGRRGROGKKGEAGROGRRGROG**LKGEQGEOGAOGIR
B-ext.	**Q**LSCTGPOA**IOGIOGIOGTOGPDGIOGIOGIOGTOGPDG**QOGTOGIKGEKGLOGLAGDH
C-ext.	NTGCYG**IOGMOGLOGAOGKDGIOGMOGLOGAOGKDG**YDGLOGPKGEPGIO
A-ext.2	EDLCRAPDGKKGEAGROGRRGRO**GLKGEQGEOGLKGEQGEO**GAOGIR
B-ext.2	**Q**LSCTGPOAIOGIOGIOGTOGPD**GQOGTOGIKGQOGTOGIK**GEKGLOGLAGDH
C-ext.2	NTGCYGIOGMOGLOGAOGKDGYD**GLOGPKGEPGLOGPKGEP**GIO
B-crt	**Q**LSCTGPOAIOGIOGIOGTOGPDGQOGTOGIKGEKGLO**GLAGDH**
A-R16A	EDLCRAPDGKKGEAG**A**OGRRGROGLKGEQGEOGAOGIR
A-R19A	EDLCRAPDGKKGEAGROG**A**RGROGLKGEQGEOGAOGIR
A-R22A	EDLCRAPDGKKGEAGROGRRG**A**OGLKGEQGEOGAOGIR
A-tRA	EDLCRAPDGKKGEAG**A**OG**A**RG**A**OGLKGEQGEOGAOGIR
C-M10D	NTGCYGIOG**D**OGLOGAOGKDGYDGLOGPKGEPGIO
C-M10N	NTGCYGIOG**N**OGLOGAOGKDGYDGLOGPKGEPGIO
C-M10L	NTGCYGIOG**L**OGLOGAOGKDGYDGLOGPKGEPGIO
C-M10F	NTGCYGIOG**F**OGLOGAOGKDGYDGLOGPKGEPGIO
C-O-A	NTGCYGI**A**GM**A**GL**A**GA**A**GKDGYDGLOGPKGEPGIO
C-O-P	NTGCYGI**P**GM**P**GL**P**GA**P**GKDGYDGLOGPKGEPGIO

"ext." represents "extended". "O" represents 2S,4R-hydroxyproline. The glutamine "Q" residue at the N-terminus of peptide C1qB and its variant peptides is 5-oxopyrrolidine-2-carboxylic acid. The colored region indicates mutation positions compared to the A, B, C peptides that modeled the native C1q. Peptides A, B, and C were designed and investigated in the reference.[12]

Table S2 Peptide sequence and molecular weight information.

Peptide	Sequences	$[M+H]^+$ expected	$[M+H]^+$ observed
A	EDLCRAPDGKKGEAGROGRRGROGLKGEQGEOGAOGIR	3990.1	3991.7
B-crt	**Q**LSCTGPOAIOGIOGIOGTOGPDGQOGTOGIKGEKGLO**GLAGDH**	4222.0	4221.1
C	NTGCYGIOGMOGLOGAOGKDGYDGLOGPKGEPGIO	3474.6	3476.5
A-R16A	EDLCRAPDGKKGEAG**A**OGRRGROGLKGEQGEOGAOGIR	3944.0	3946.0
A-R19A	EDLCRAPDGKKGEAGROG**A**RGROGLKGEQGEOGAOGIR	3944.0	3946.0
A-R22A	EDLCRAPDGKKGEAGROGRRG**A**OGLKGEQGEOGAOGIR	3944.0	3945.9
A-tRA	EDLCRAPDGKKGEAG**A**OG**A**RG**A**OGLKGEQGEOGAOGIR	3773.9	3775.9
C-M10D	NTGCYGIOG**D**OGLOGAOGKDGYDGLOGPKGEPGIO	3456.6	3458.6
C-M10N	NTGCYGIOG**N**OGLOGAOGKDGYDGLOGPKGEPGIO	3455.6	3456.6
C-M10L	NTGCYGIOG**L**OGLOGAOGKDGYDGLOGPKGEPGIO	3454.6	3456.6
C-M10F	NTGCYGIOG**F**OGLOGAOGKDGYDGLOGPKGEPGIO	3488.6	3490.6
A-ext.	EDLCRAPDGKKGEAGROGRRGROGKKGEAGROGRRGROGLKGEQGEOGAOGIR	5622.9	5625.0
B-ext.	QLSCTGPOAIOGIOGIOGTOGPDGIOGIOGIOGTOGPDGQOGTOGIKGEKGLOGLAGDH	5607.5	5610.7
C-ext.	NTGCYGIOGMOGLOGAOGKDGIOGMOGLOGAOGKDGYDGLOGPKGEPGIO	4881.2	4875.4
A-ext.2	EDLCRAPDGKKGEAGROGRRGROGLKGEQGEOGLKGEQGEOGAOGIR	4941.5	4943.4
B-ext.2	QLSCTGPOAIOGIOGIOGTOGPDGQOGTOGIKGQOGTOGIKGEKGLOGLAGDH	5087.6	5088.5
C-ext.2	NTGCYGIOGMOGLOGAOGKDGYDGLOGPKGEPGLOGPKGEPGIO	4322.0	4323.0
C-O-A	NTGCYGIAGMAGLAGAAGKDGYDGLOGPKGEPGIO	3307.0	3306.6
C-O-P	NTGCYGIPGMPGLPGAPGKDGYDGLOGPKGEPGIO	3409.8	3410.8

"ext." represents "extended". "O" represents 2S,4R-hydroxyproline. The glutamine "Q" residue at the N-terminus of peptide C1qB and its variant peptides is 5-oxopyrrolidine-2-carboxylic acid.

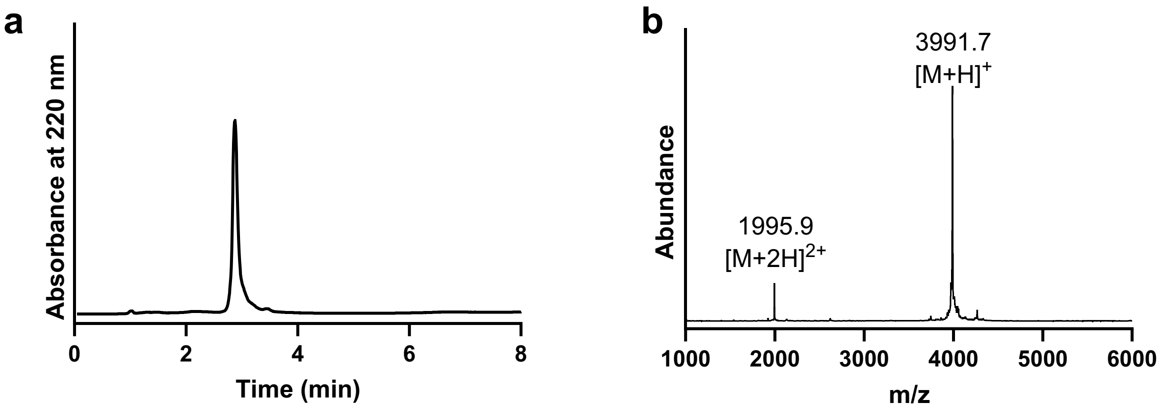

Supplementary Figure S1. a) liquid chromatography of pure peptide A. b) mass spectrum of pure peptide A.

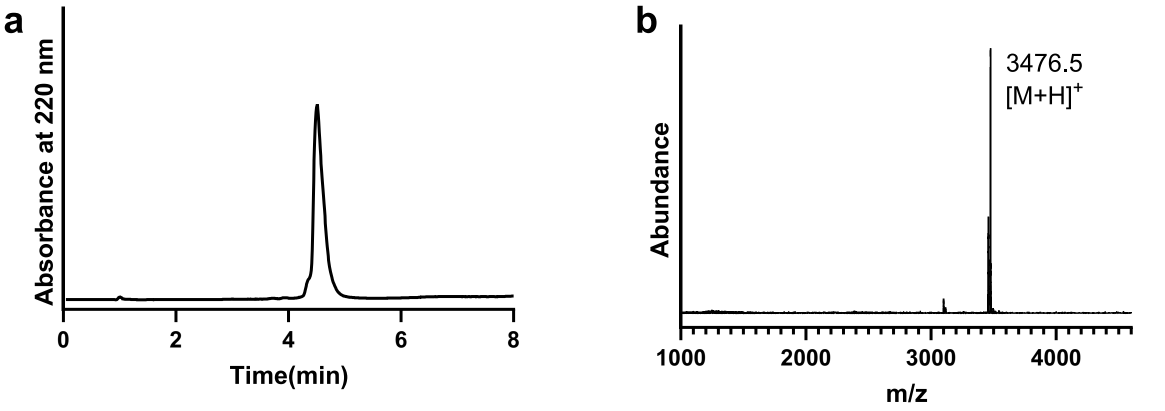

Supplementary Figure S2. a) liquid chromatography of pure peptide C. b) mass spectrum of pure peptide C.

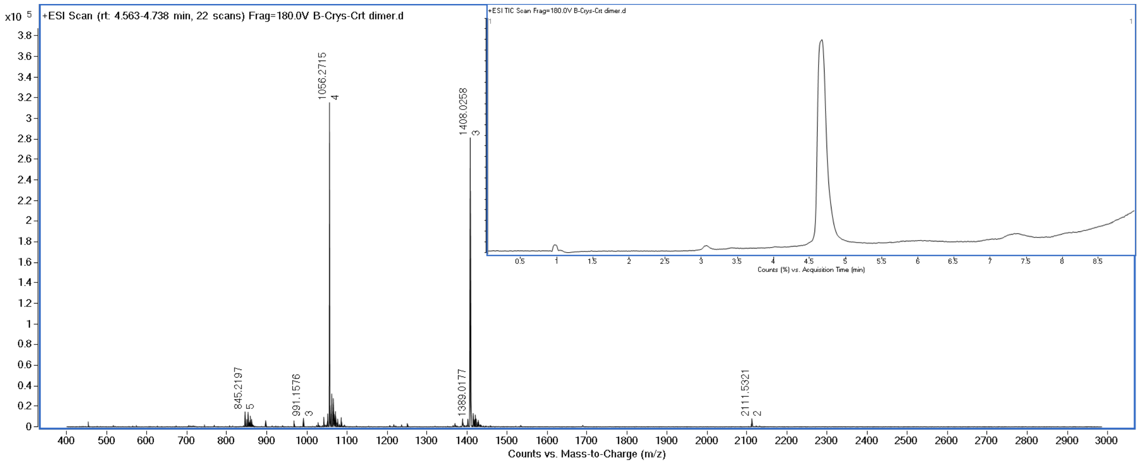

Supplementary Figure S3. LC-MS characterization of C1qB-Crt peptide.

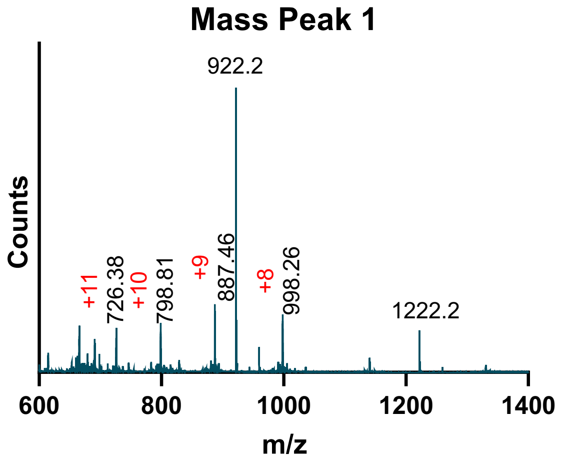

Supplementary Figure S4. Mass spectrometry of the first peak in the liquid chromatography displayed in Figure 1e. The molecular weight observed corresponds to the A-A dimer. The peaks of 922.2 and 1222.2 Da are the calibration peaks of protein standards.

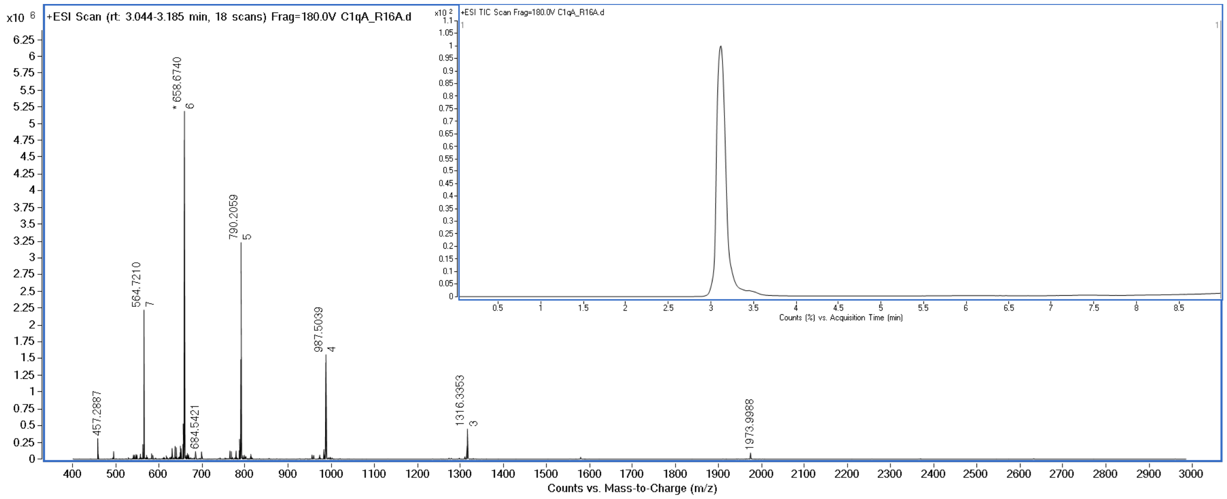

Supplementary Figure S5. LC-MS of C1qA-R16A peptide.

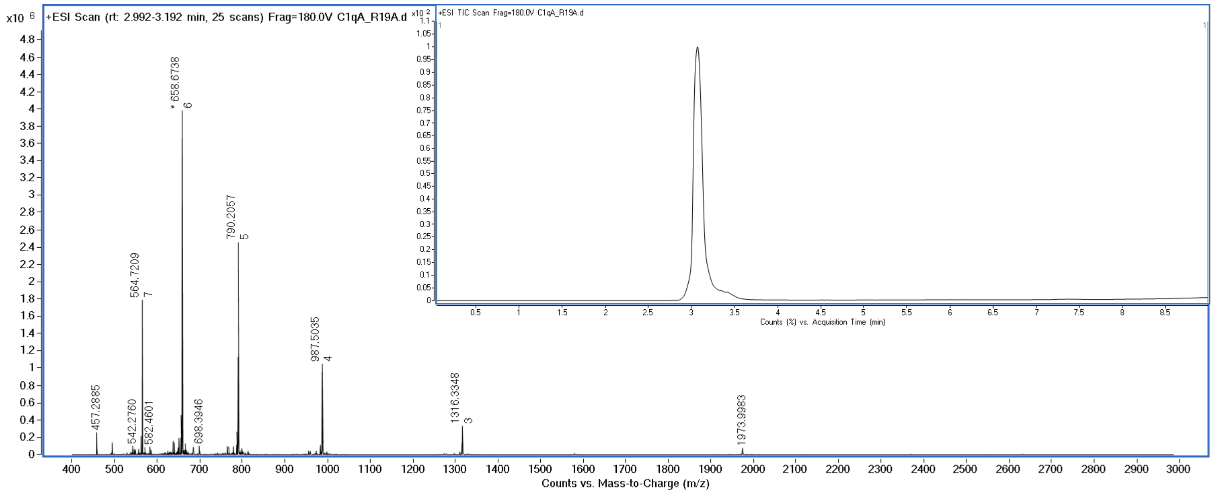

Supplementary Figure S6. LC-MS of C1qA-R19A peptide.

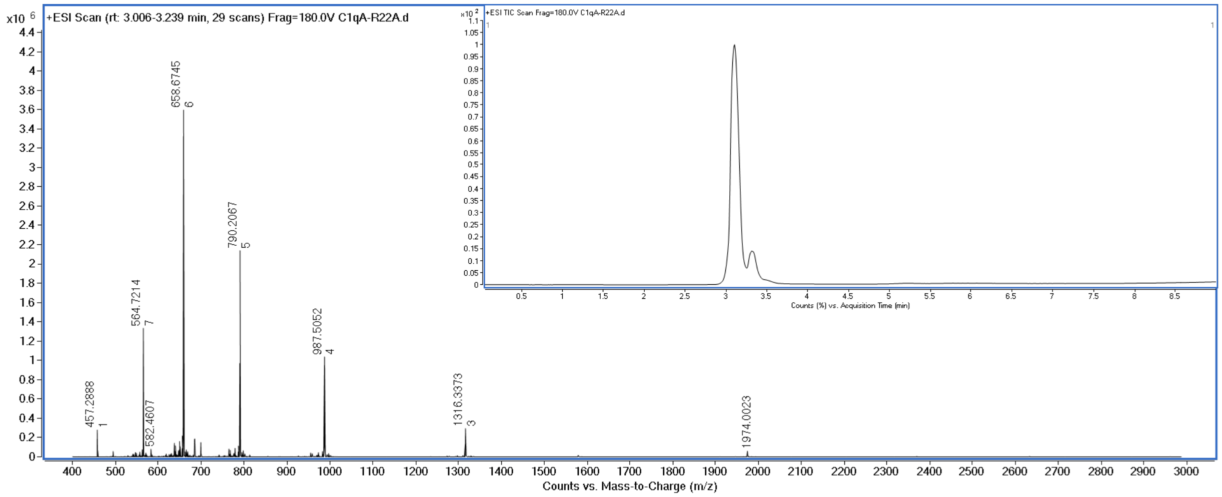

Supplementary Figure S7. LC-MS of C1qA-R22A peptide.

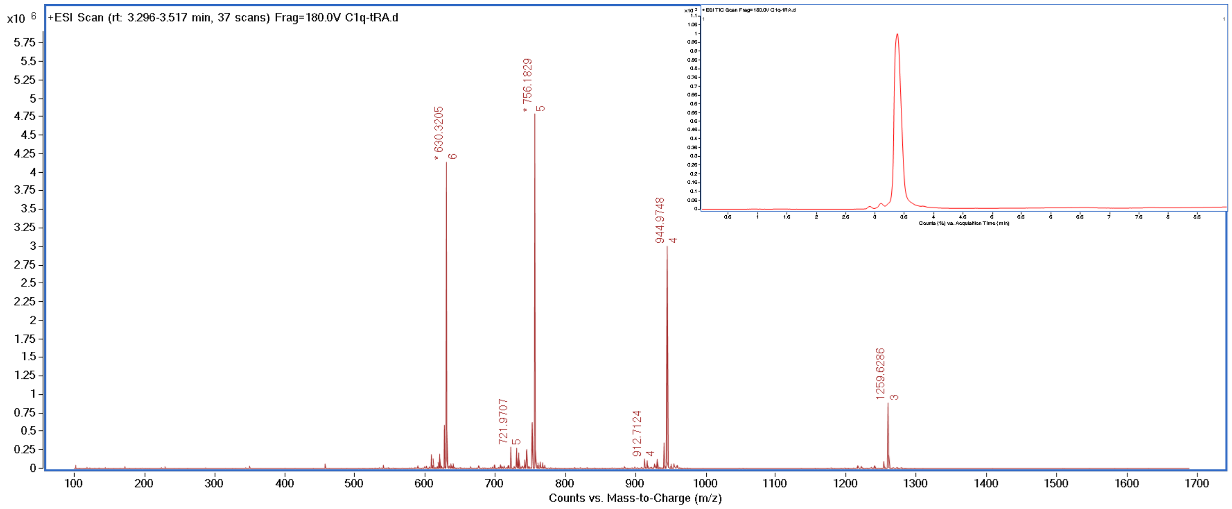

Supplementary Figure S8. LC-MS of C1qA-tRA peptide.

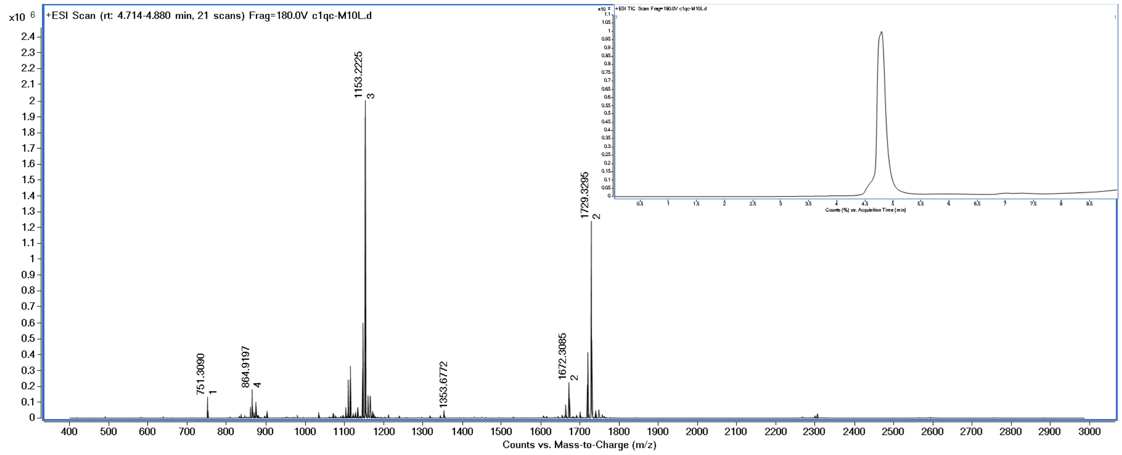

Supplementary Figure S9. LC-MS characterization of C1qC-M10L peptide.

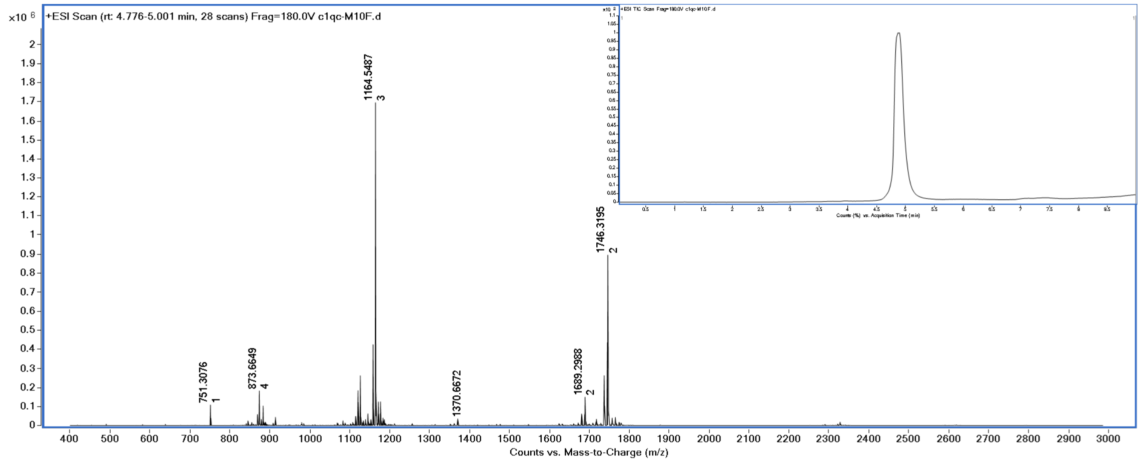

Supplementary Figure S10. LC-MS characterization of C1qC-M10F peptide.

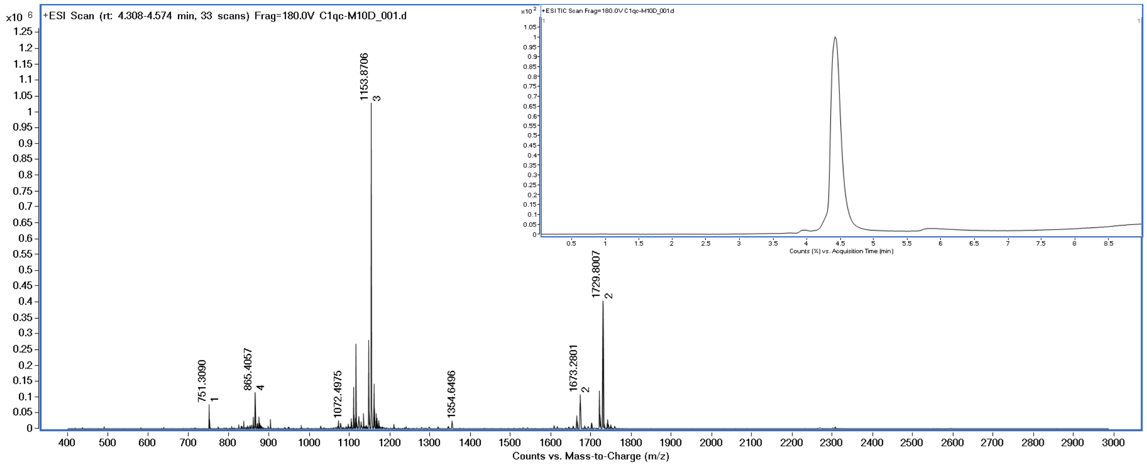

Supplementary Figure S11. LC-MS characterization of C1qC-M10D peptide.

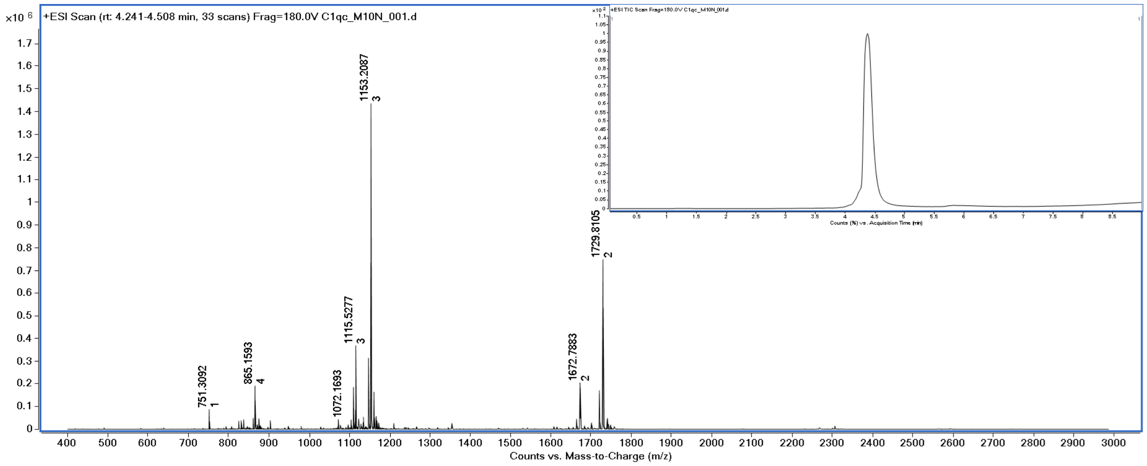

Supplementary Figure S12. LC-MS characterization of C1qC-M10N peptide.

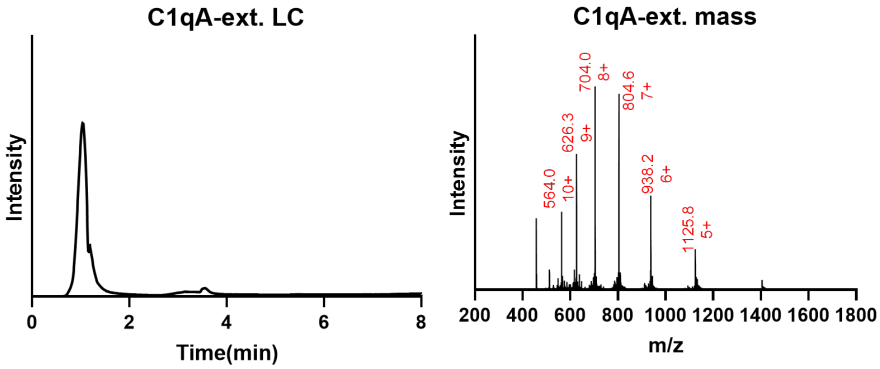

Supplementary Figure S13. LC-MS characterization of A-ext. peptide.

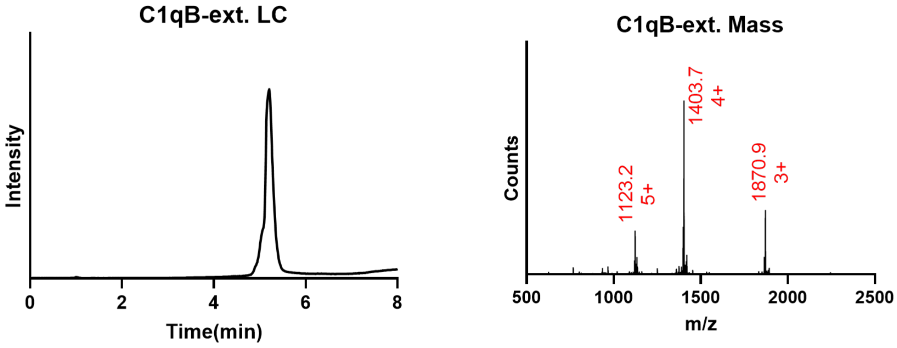

Supplementary Figure S14. LC-MS characterization of B-ext. peptide.

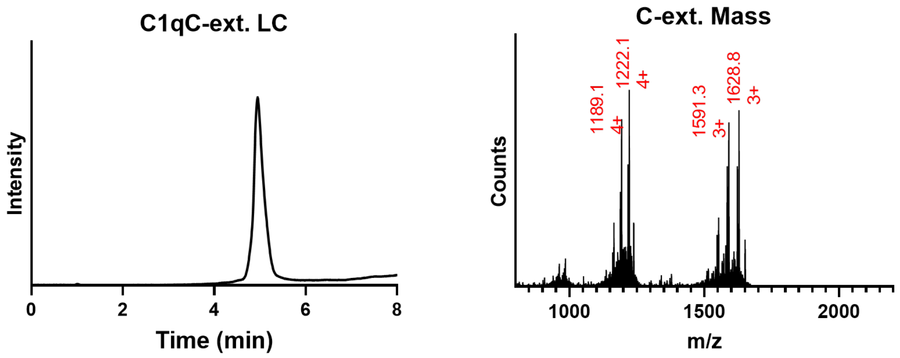

Supplementary Figure S15. LC-MS characterization of C-ext. peptide.

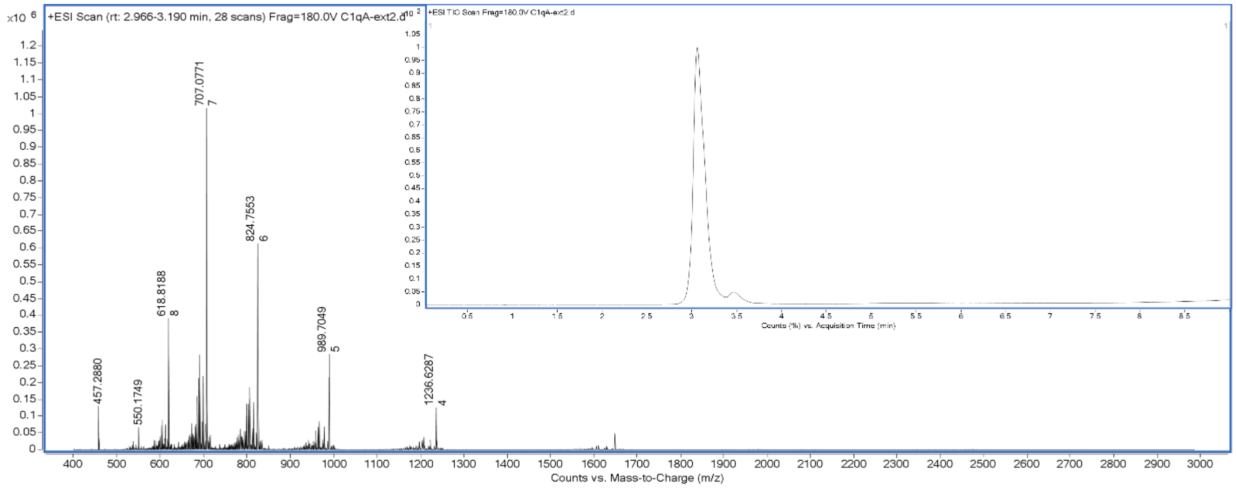

Supplementary Figure S16. LC-MS characterization of A-ext.2 peptide.

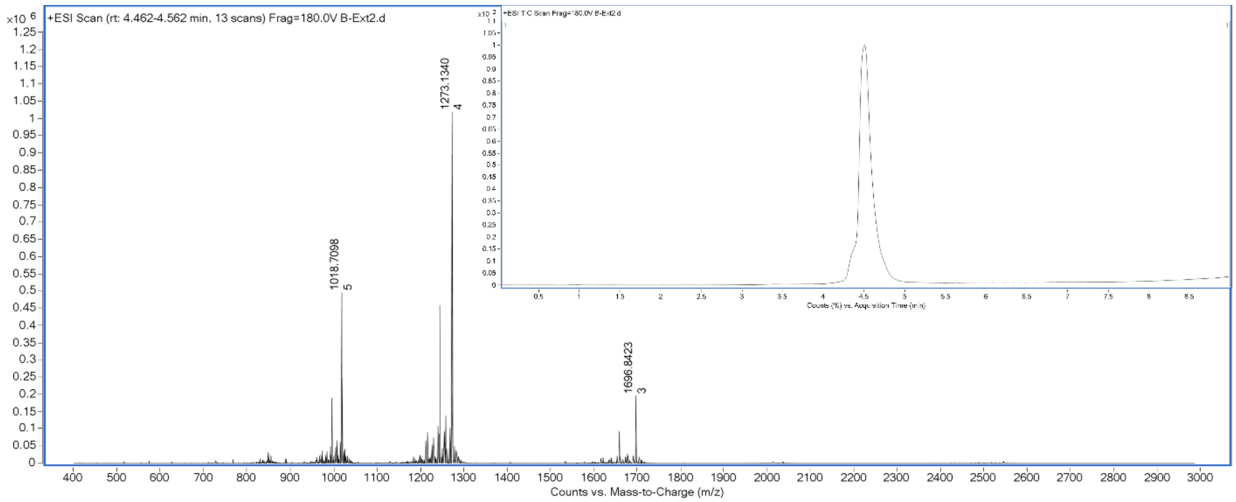

Supplementary Figure S17. LC-MS characterization of B-ext.2 peptide.

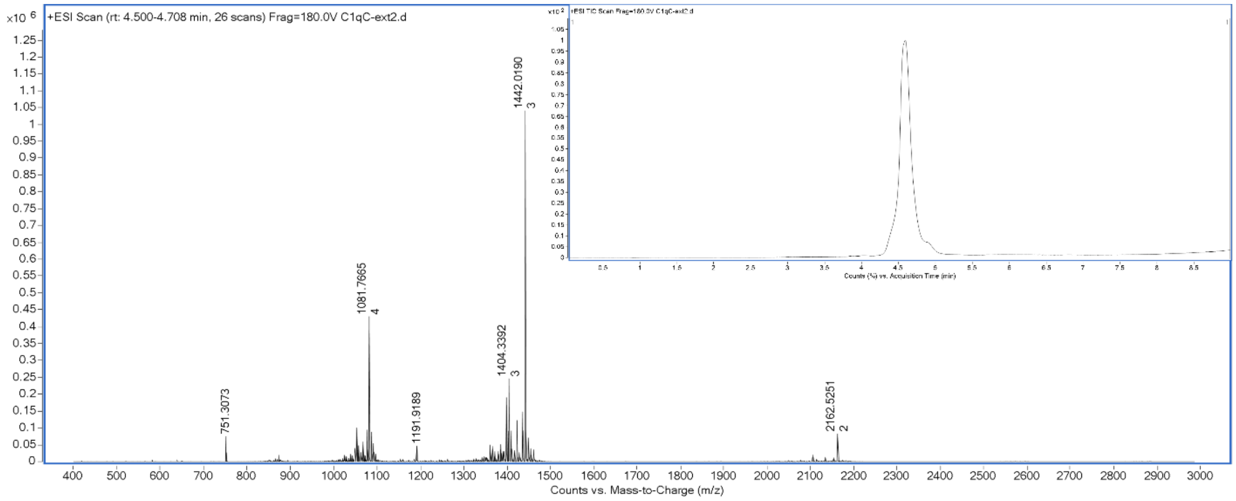

Supplementary Figure S18. LC-MS characterization of C-ext.2 peptide.

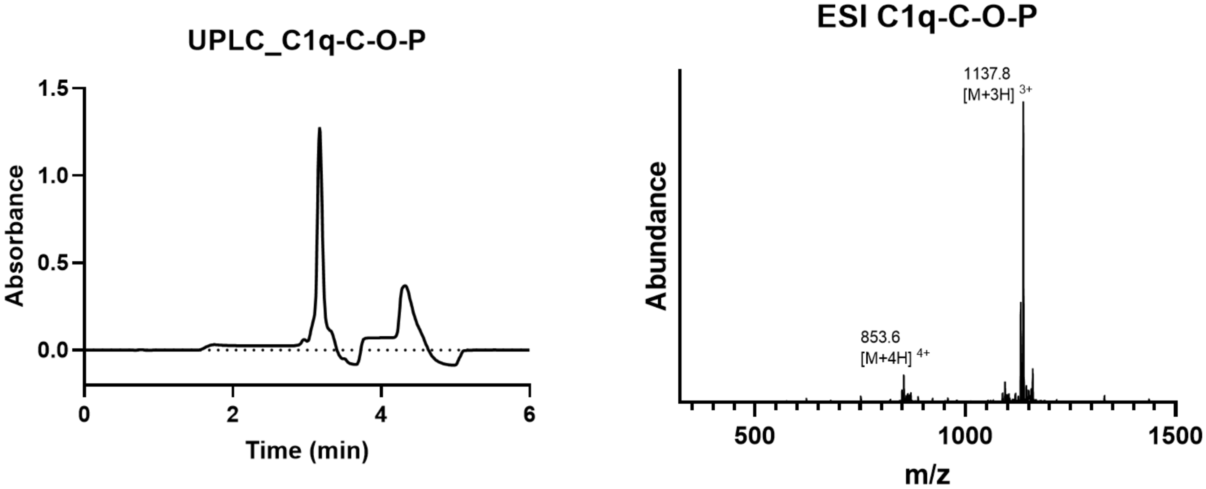

Supplementary Figure S19. UPLC and ESI characterization of C-O-P peptide.

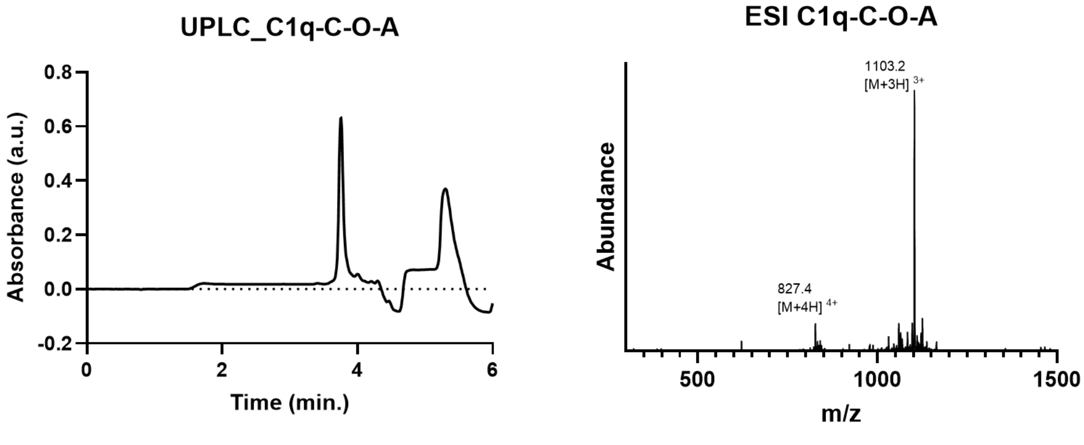

Supplementary Figure S20. UPLC and ESI characterization of C-O-P peptide.

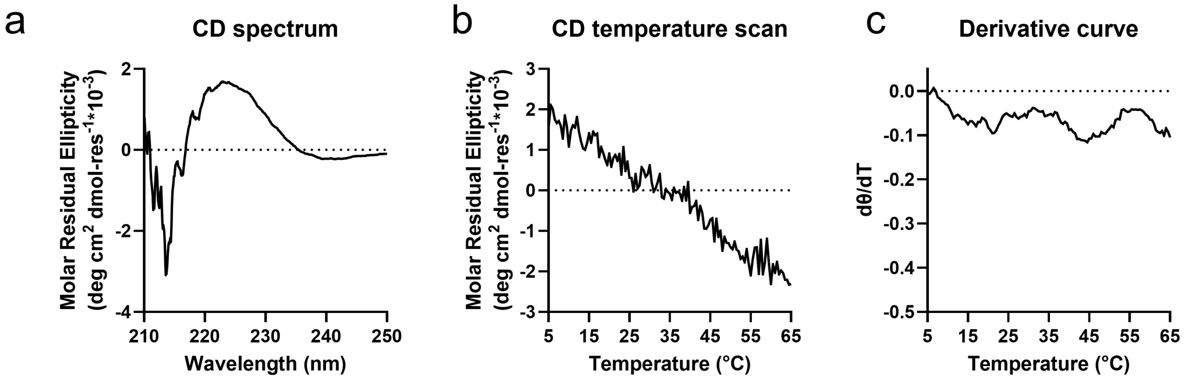

Supplementary Figure S21. CD characterization of trimeric peptide mixture of A-ext., B-ext., and C-ext. a) CD spectrum scanning. b) CD variable-temperature scanning. c) first-order derivative of the variable-temperature scanning displayed in b).

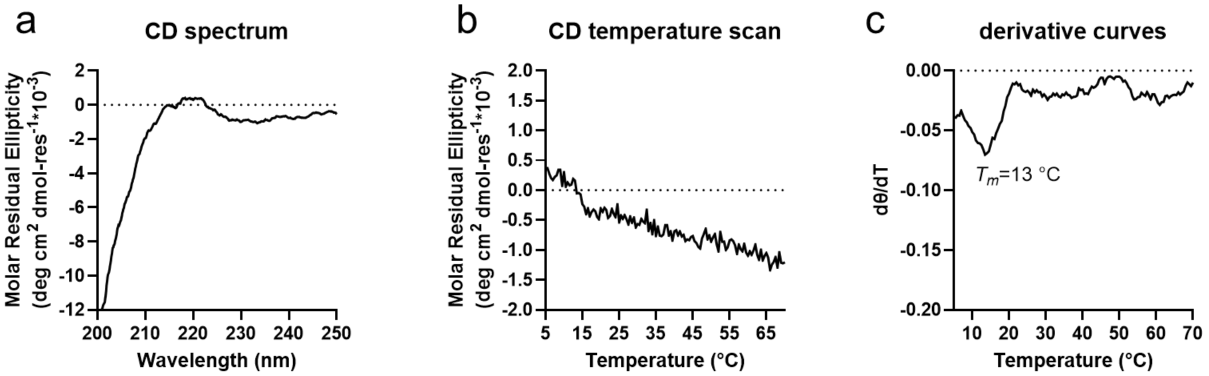

Supplementary Figure S22. CD characterization of trimeric peptide mixture of A-ext.2, B-ext.2, and C-ext.2. a) CD spectrum scanning. b) CD variable-temperature scanning. c) first-order derivative of the variable-temperature scanning displayed in b).

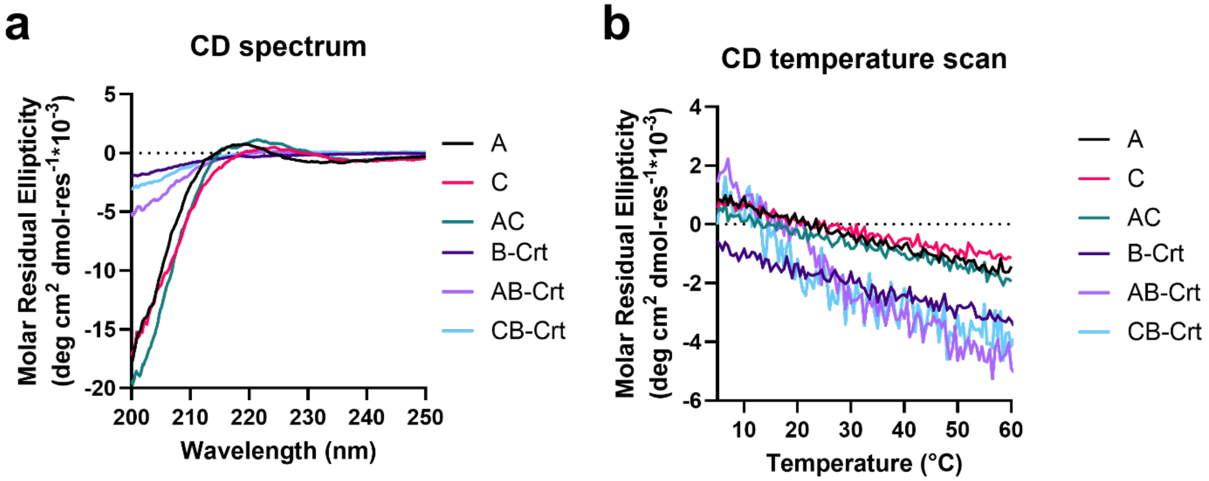

Supplementary Figure S23. CD characterization of monomeric peptide and binary peptide mixtures of C1q A, B-Crt, and C peptides. a) CD spectra. b) variable temperature-wavelength scan.

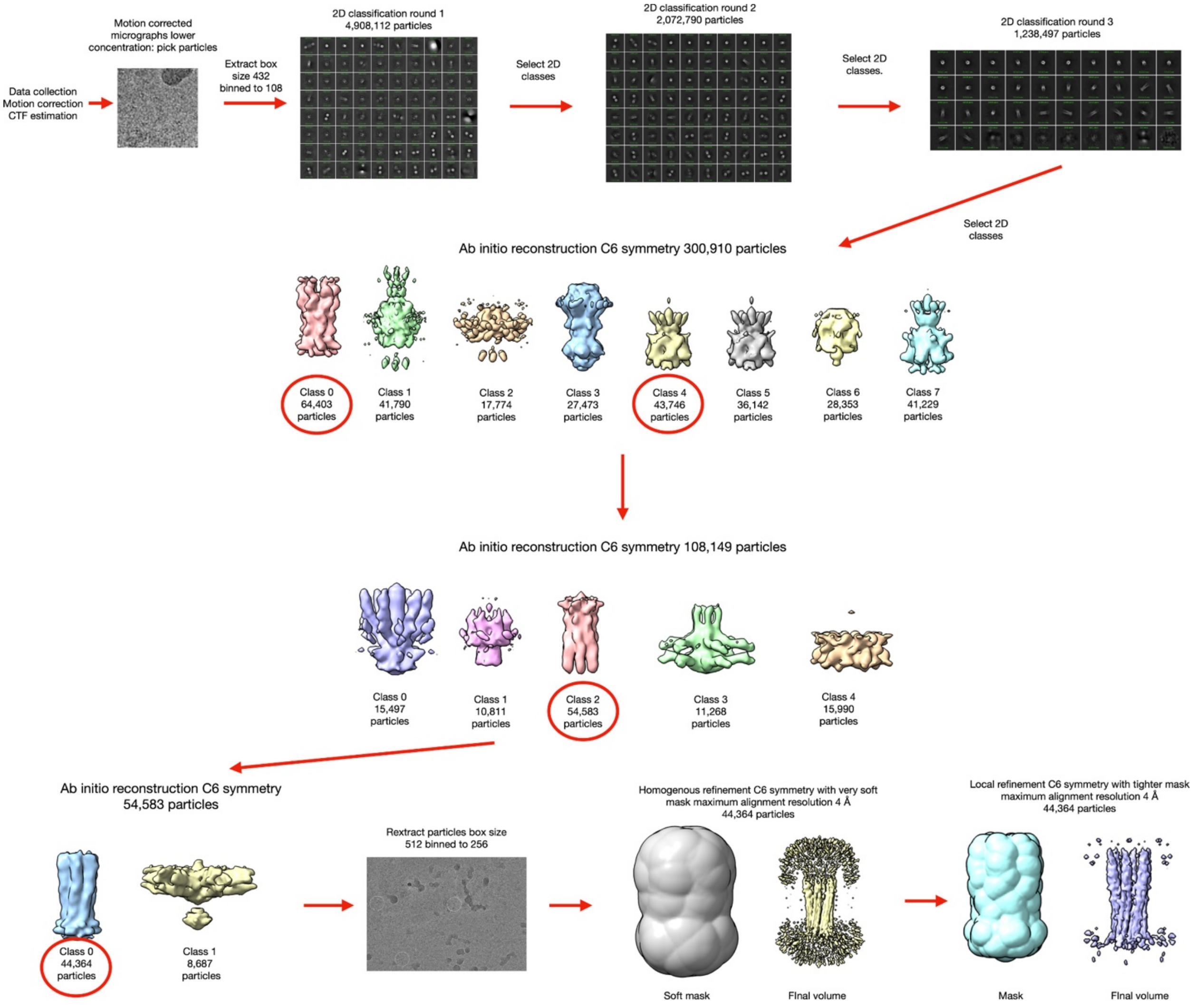

Supplementary Figure S24. Workflow for cryo-EM reconstruction of the larger C1q cys crt density map.

Table S3. Cryo-EM map and model statistics

	C1q cys crt Narrow region map + model	C1q cys crt EMReady narrow region map +model	C1q cys crt low resolution full map +model	C1q cys crt low resolution EMReady full map + model	C-M10N just full density map	C-M10F just full density map	hC1q assembly
PDB ID	9C9L	9C9L	9C9U	9C9U	N/A	N/A	N/A
EMDB ID	EMD-45363 Additional map	EMD-45363 Primary map	EMD-45371 Primary map	EMD-45371 Additional map	EMD-45373	EMD-45365	EMD-45372
map:map 0.143 FSC	3.7	N/A	3.8	N/A	4.5	4.5	3.6[#]
Map:model 0.5 FSC	3.9	3.5	5.4	5.2	N/A	N/A	N/A
Clash score	9.15	9.15	19.72	19.72	N/A	N/A	N/A
CC (mask)	0.77	0.83	0.78	0.62	N/A	N/A	N/A
MolProbity Score	1.49	1.49	2.36	2.36	N/A	N/A	N/A
Ramachandran favored (%)	100	100	89.29	89.29	N/A	N/A	N/A
Ramachandran allowed (%)	0	0	10.71	10.71	N/A	N/A	N/A
Ramachandran outliers (%)	0	0	0	0	N/A	N/A	N/A
Bond length RMSD (Å)	0.007	0.007	0.004	0.004	N/A	N/A	N/A
Bond Angle RMSD (°)	1.258	1.258	0.987	0.987	N/A	N/A	N/A
Rotamer outliers (%)	0	0	0	0	N/A	N/A	N/A
# particles	374,558	374,558	44,364	44,364	138,738	92,001	79,400
pixel size	1.34 (0.67 2x bin)	1 (EMReady)	1.34 (0.67 2x bin)	1 (EMReady)	1.34 (0.67 2x bin)	1.34 (0.67 2x bin)	1.34 (0.67 2x bin)

*The map model FSC crossed 0.5 FSC twice once at 8.1 Å and again at 4.7 Å. Given the quality of the map and the fact that we could see separated PPII chains (~5 Å apart) we took the 4.7 Å estimate.

[#]The calculated 0.143 Map:Map FSC is likely an overestimate. We think that the resolution of the map is closer to ~4.5 Å.

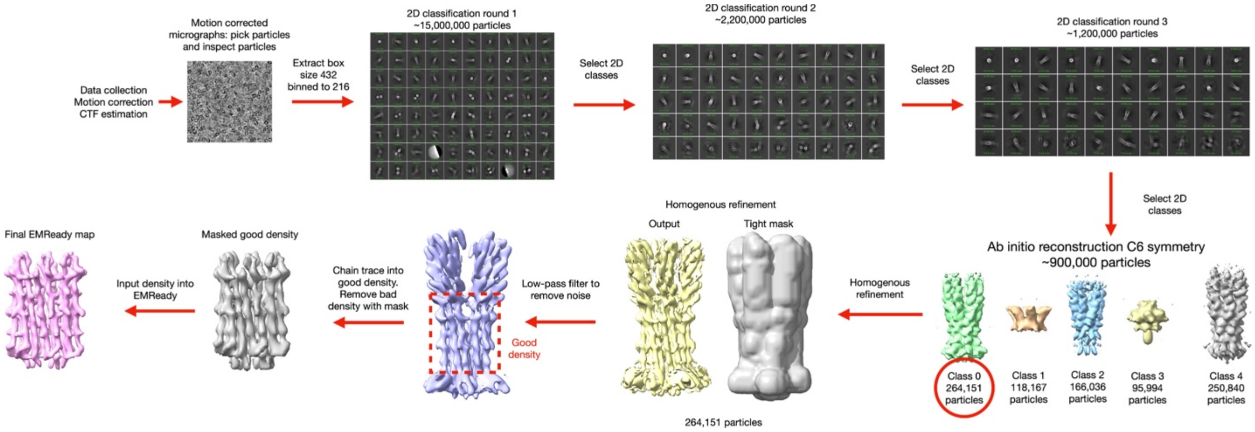

Supplementary Figure S25. The general workflow for the processing of cryo-EM data in this paper is shown. In general, all structures determined were processed in this way with differences described in methods.

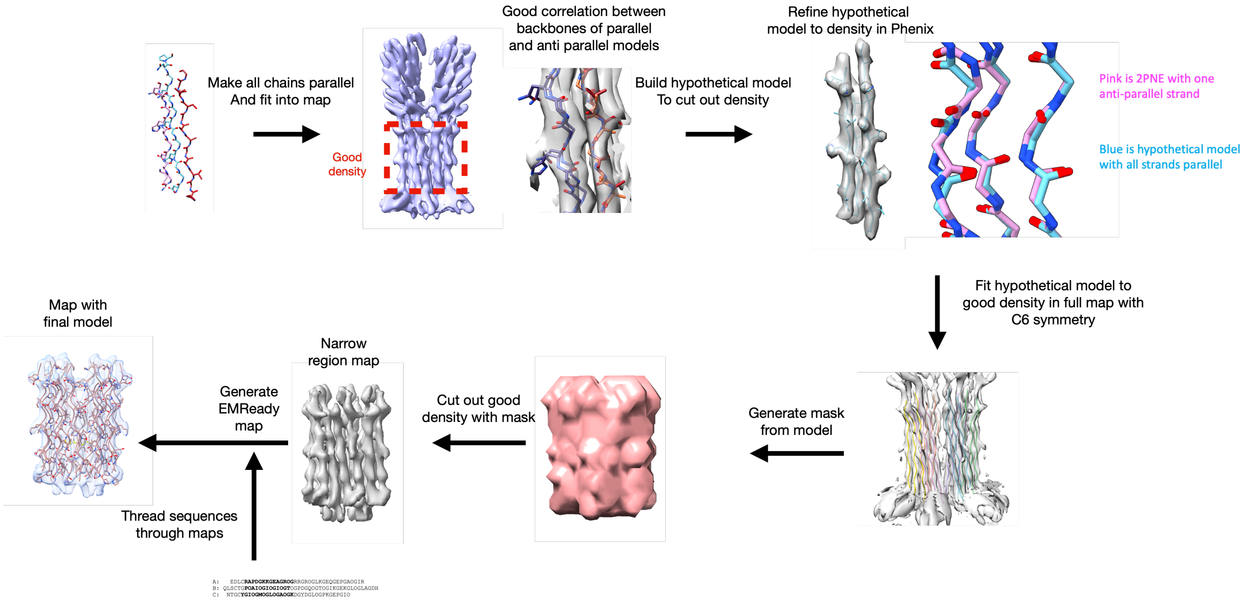

Supplementary Figure S26. Description of atomic modeling of narrow region. A general workflow is shown for how the model of the C1q cys crt narrow region was built.

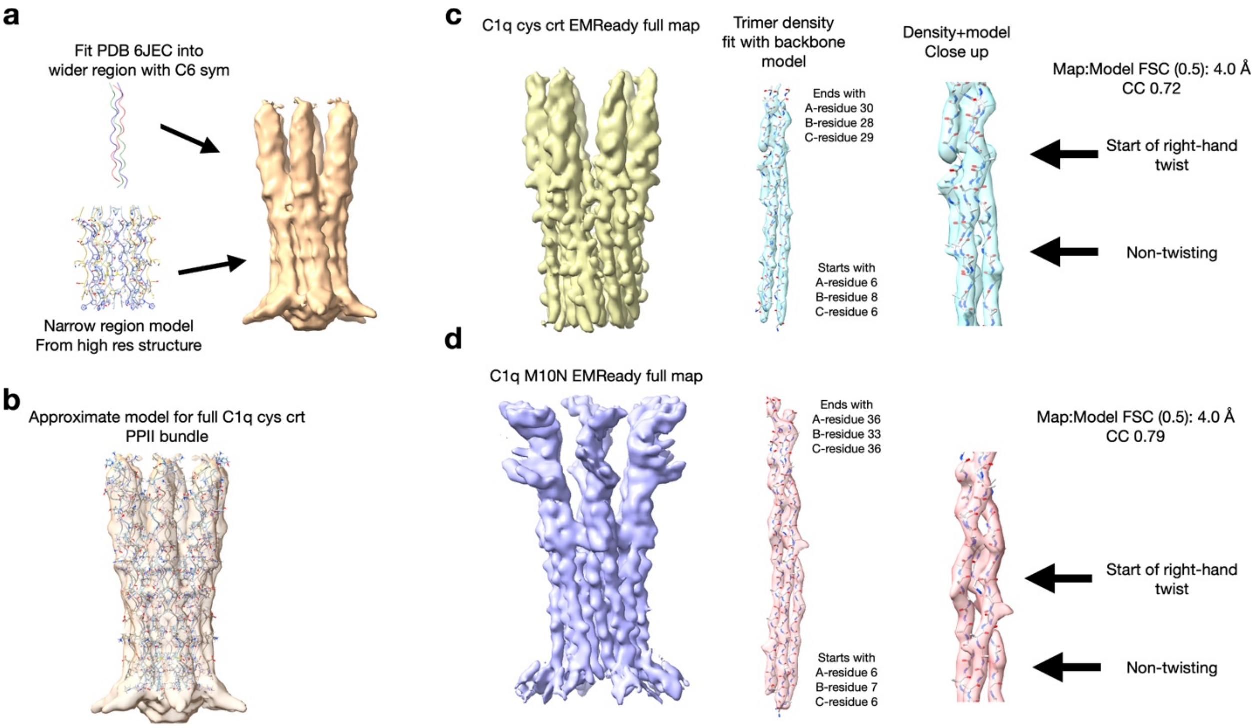

Supplementary Figure S27. Description of atomic modeling of the full C1q assembly and fit and comparisons into the EMReady density maps. **a)** Depiction of how the narrow region model and PDB 6JEC were fit into the low-resolution density map. **b)** Fit of the longer C1q cys crt into the low-resolution density map. **c)** EMReady map of the full C1q cys crt structure is shown of the left. In the middle and on the right is a single PPII trimer model fit into its corresponding map. **d**) The EMReady map of the full C-M10N structure is shown on the left. In the middle and on the right is a single PPII trimer model fit into its corresponding C-M10N map.

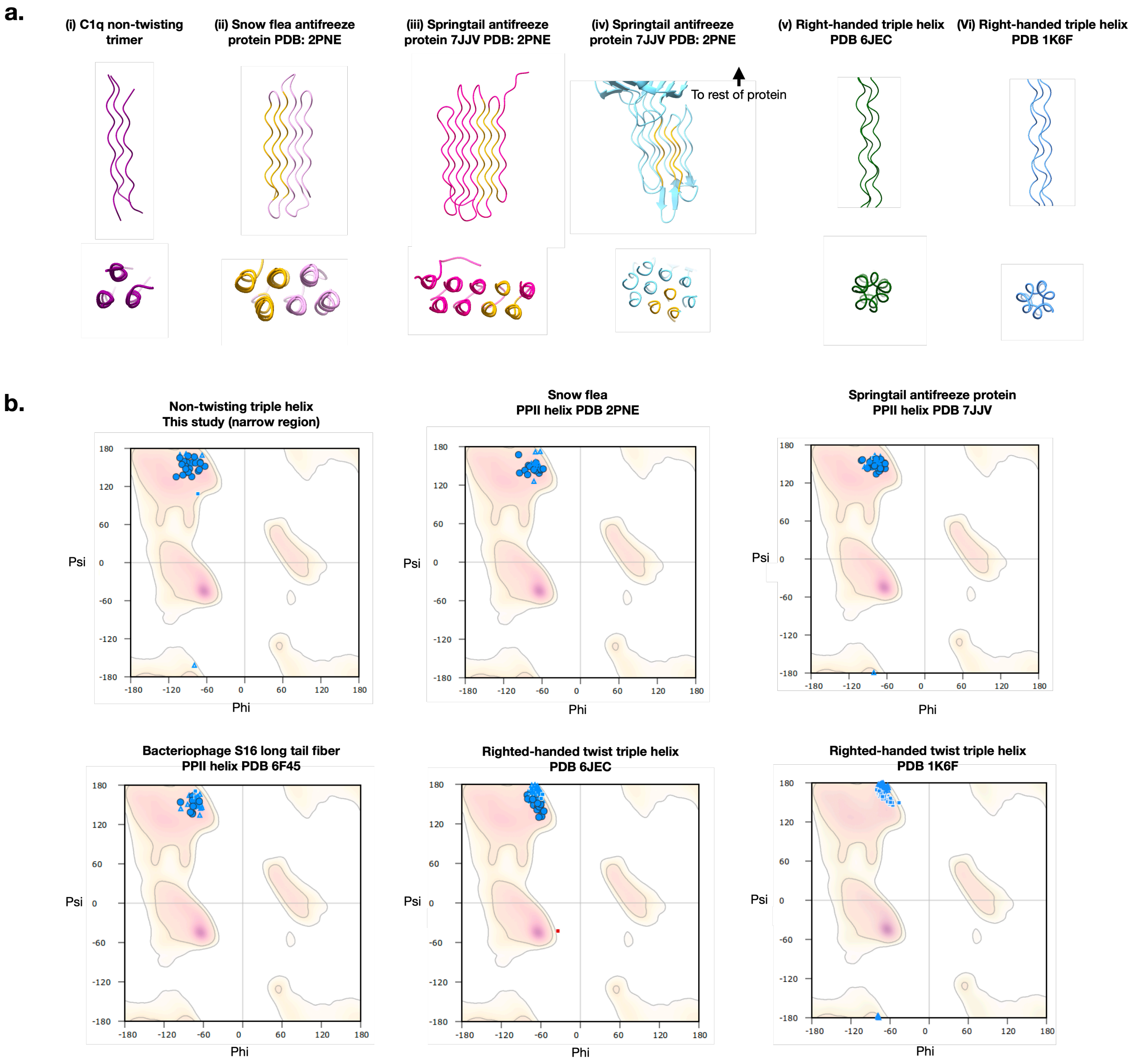

Supplementary Figure S28. Comparison of models and Ramachandran plots of different PPII helices. a) Models of the C1q non-twisting region (i), several single protein chain non-twisting PPII helices (ii – iv), and two canonical right-handed triple helices (v-vi). For the single chain PPII helices the gold residues correspond to a three strand section of PPII helices that conformationally resembled the C1q non-twisting triple helix. **b)** Ramachandran plots of the various PPII assemblies from a). For the single protein chain PPII assemblies a stretch of amino acids corresponding to three interfacing PPII strands was chosen similar as shown in gold in a). The loop regions between these continuous PPII strands were removed.

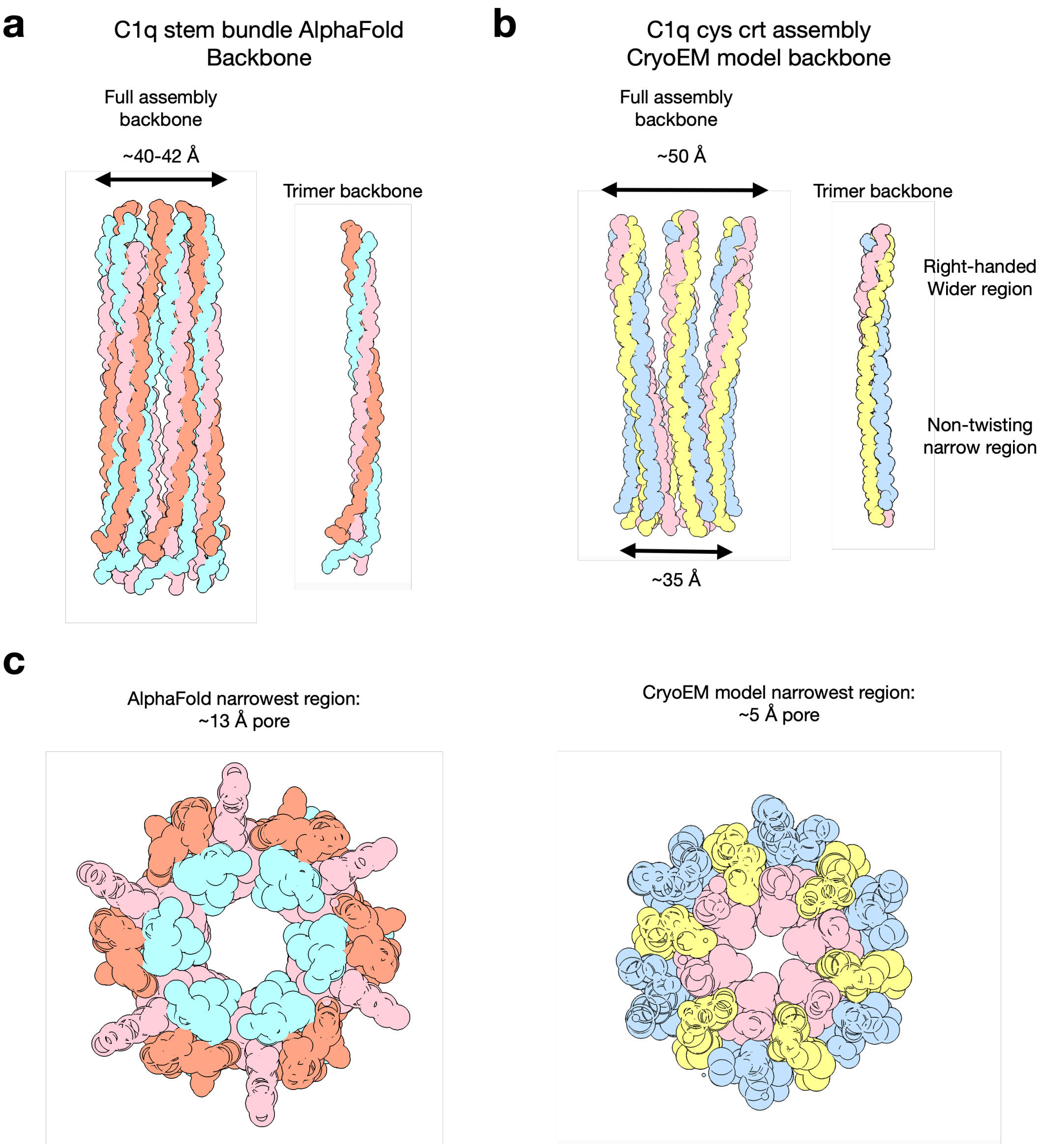

Supplementary Figure S29. Comparison between an AlphaFold3 predicted model of the C1q assembly and the C1q cys crt model from cryoEM data. **a)** The left image shows the atomic backbone for the AlphaFold predicted C1q assembly. The right image shows the backbone of a single triple-helical trimer of the assembly predicted by alphafold. **b)** The left image shows the model backbone for the C1q cys crt peptide assembly from the lower resolution full cryo-EM structure. The right image shows a trimer backbone with both the non-twisting PPII helix region as well as the right-handed twisting PPII region. **c)** The left image is a view through the pore of the AlphaFold predicted assembly model showing the ~13 Å wide pore. The right image shows the same view of the C1q cys crt assembly model determined from the cryo-EM data.

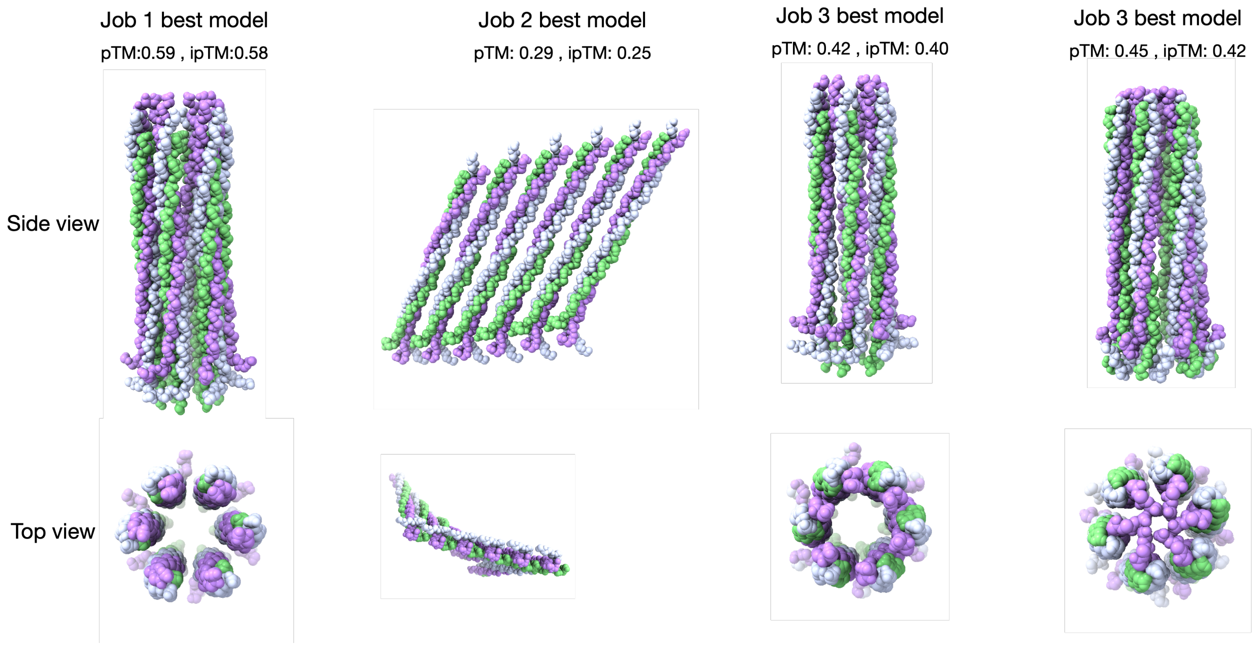

Supplementary Figure S30. Comparison of the top scoring models generated from four independent AlphaFold3 jobs on the AlphaFold Server. The term pTM stands for the predicted template modeling score. The term ipTM stands for the interface template modeling score. These are metric used to measure the quality of the overall fold of the complex and the subunit positions in the complex respectively. For pTM greater than 0.5 the overall fold might be similar to the true structure. For ipTM less than 0.6 the prediction is likely failed or incorrect.

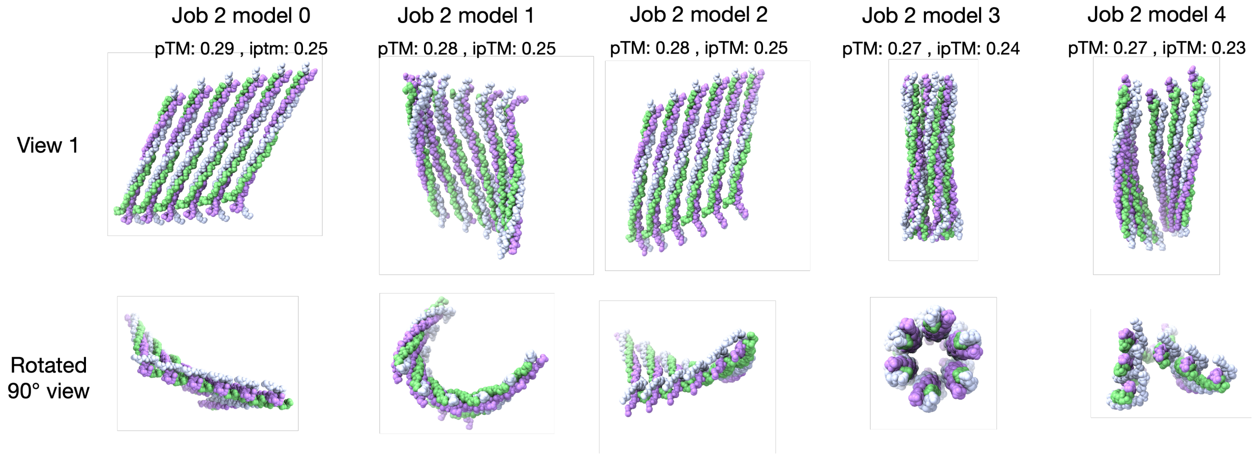

Supplementary Figure S31. Comparison of the five models generated from AlphaFold modeling job 2.

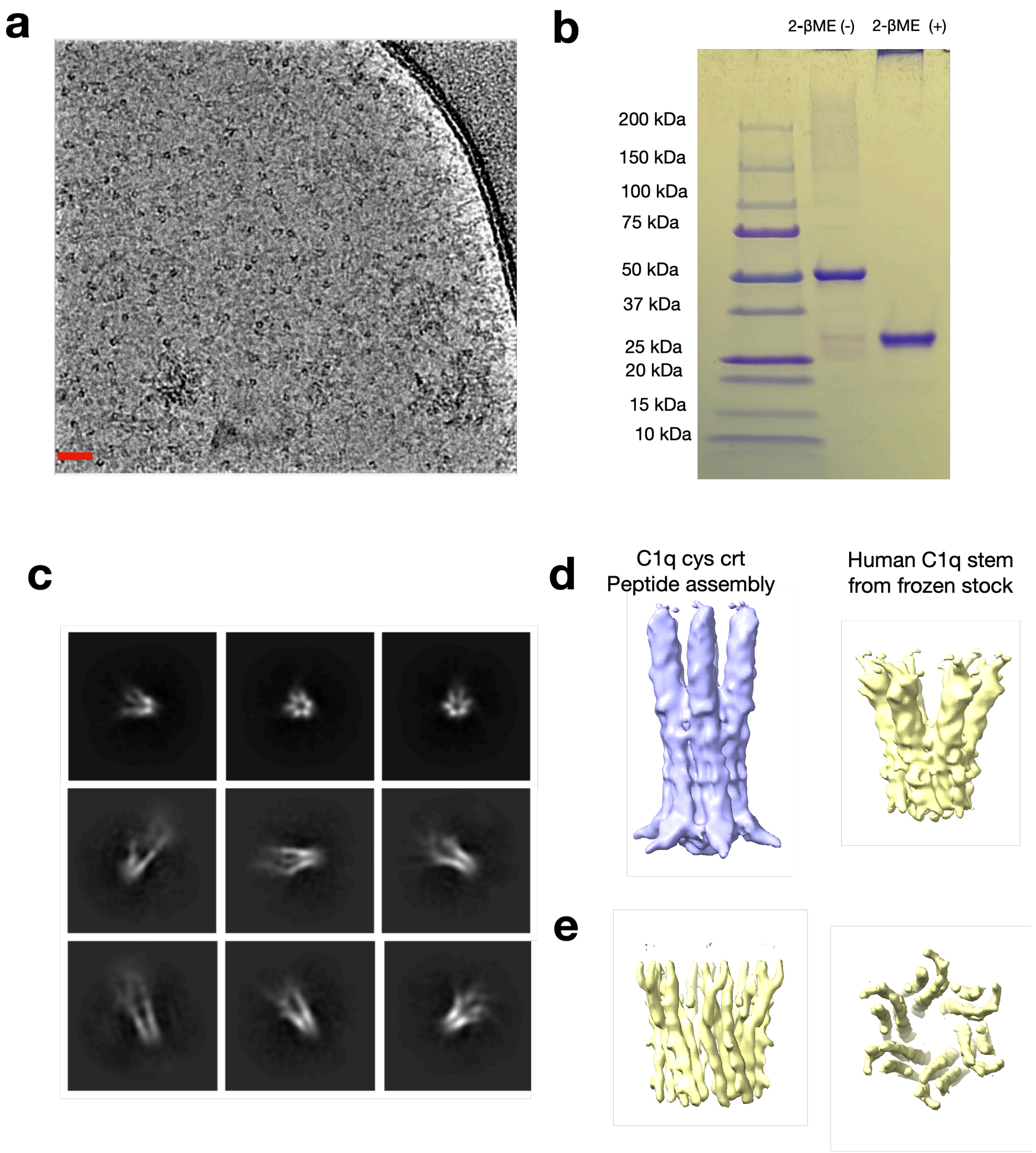

Supplementary Figure S32. Cryo-EM structural analysis of the human C1q collagenous stem bundle. a) Cryo-electron micrograph of the hC1q sample. b) SDS-PAGE analysis of the hC1q assembly in the presence, "2-BME(+)", and absence, "2-BME(-)", of reducing agent 2-β-mercaptoethanol. c) 2D-classes of the hC1q stem bundles. e) Comparison in the density maps of the full C1q cys crt peptide assembly (left) and the hC1q stem bundle (right). e) Side view (left) and top view (right) of the resolvable PPII density for the hC1q stem bundle. f) Close-up of the PPII trimer for the hC1q stem bundle showing both the non-twisting region and the region with a right-handed twist.

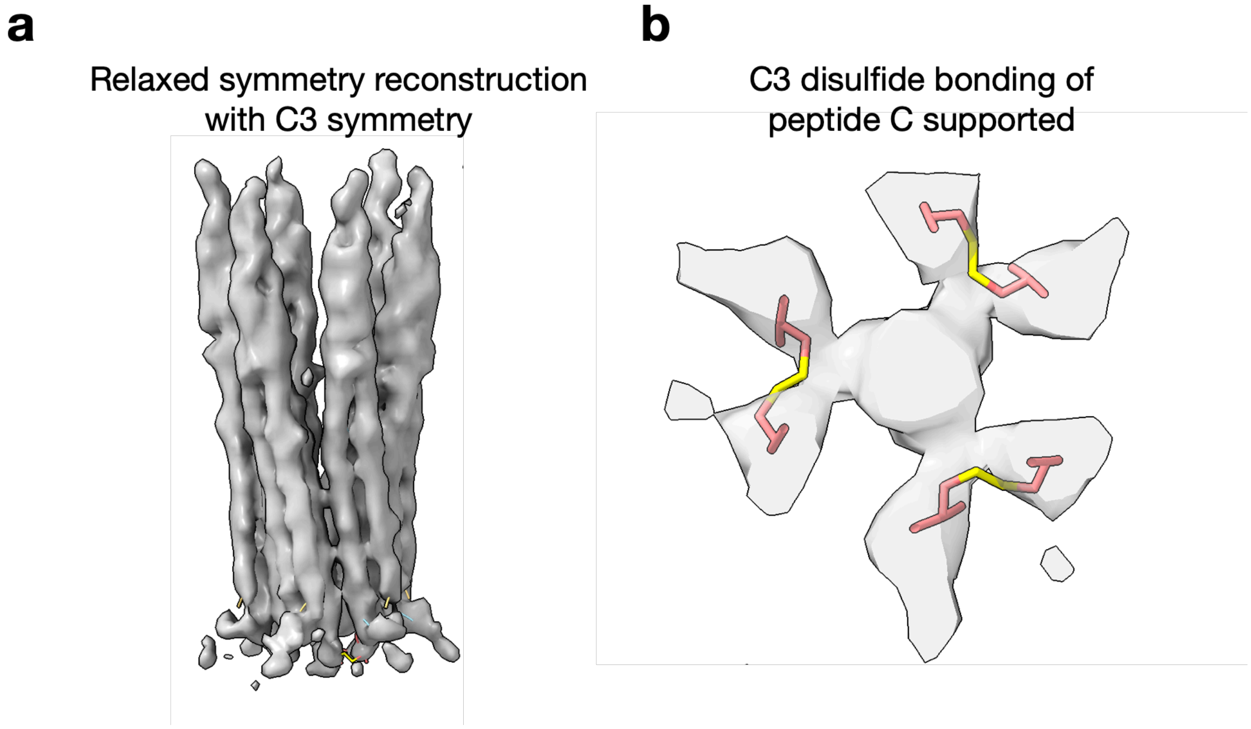

Supplementary Figure S33. Likely disulfide bonding scheme in the C1q stem. a) The volume from a relaxed symmetry reconstruction where the C6 symmetry reconstruction and particles were subject to local refinement imposing C3 symmetry. b) Density region of the C3 reconstruction map that appears to be in a plausible location for peptide C disulfide bonding.

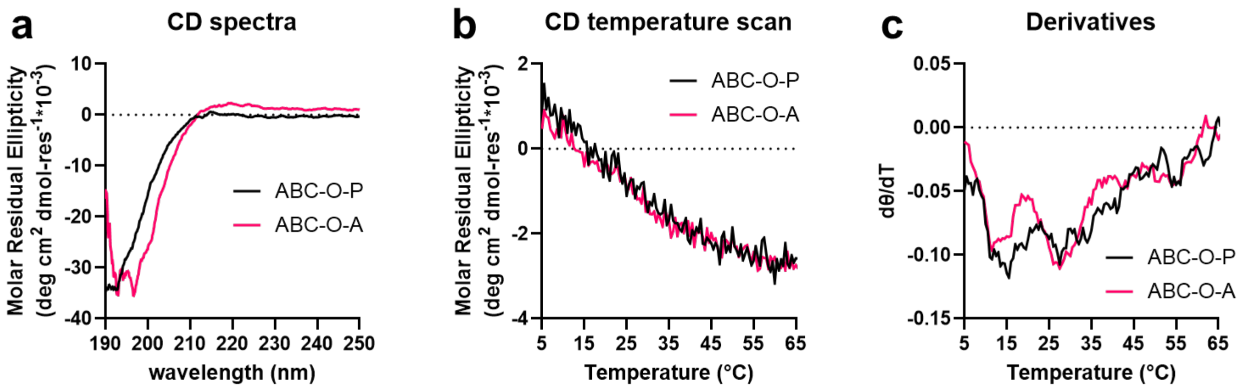

Supplementary Figure S34. CD characterization of trimeric peptide mixtures of C1q A, B, C-O-P and C-O-A peptides. a) CD spectra. b) variable temperature-wavelength scan and c) first-order derivative of the melting curves presented in b).

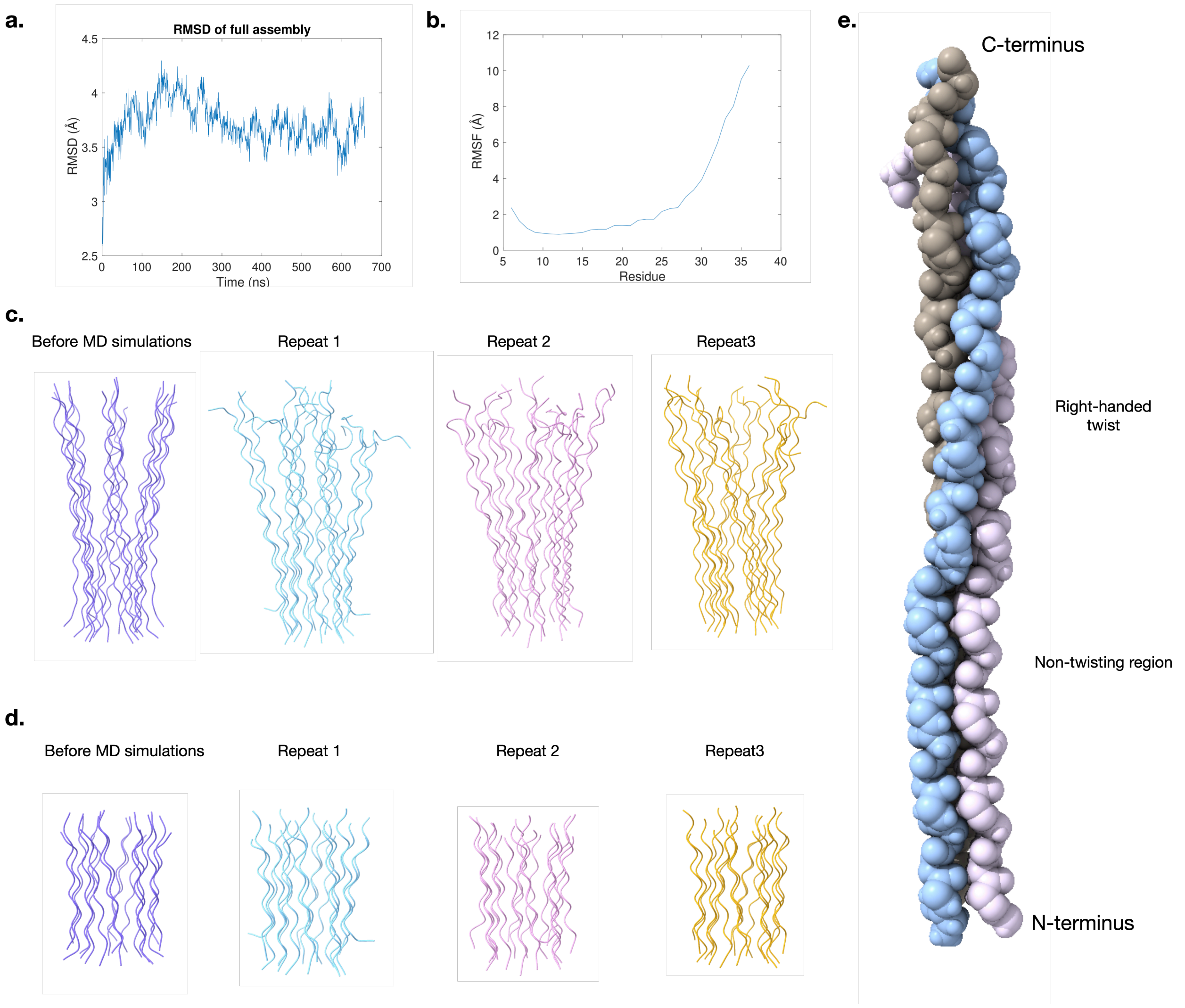

Supplementary Figure S35. Stability of the C1q cys crt peptide assembly after MD simulations. a) Plot showing the RMSD of the full assembly versus time during the duration of the MD simulations. **b)** Plot showing the time-averaged root mean squared fluctuations (RMSF) for each residue in the model's position. **c)** From left to right the models of the full C1q cys crt complex are shown before the MD simulations and then after the final steps for each of the three repeats. There is significant variability in positioning and conformation of the assemblies at the C-terminal end likely due to the limited resolution of the cryo-EM map resulting in an imperfect model. **d)** The models of the C1q cys crt narrow region before MD simulations as well as the final outputs after each simulation repeat are shown. **e)** Spherical atom representation of a single triple helix model from one of final outputs of the MD simulations.

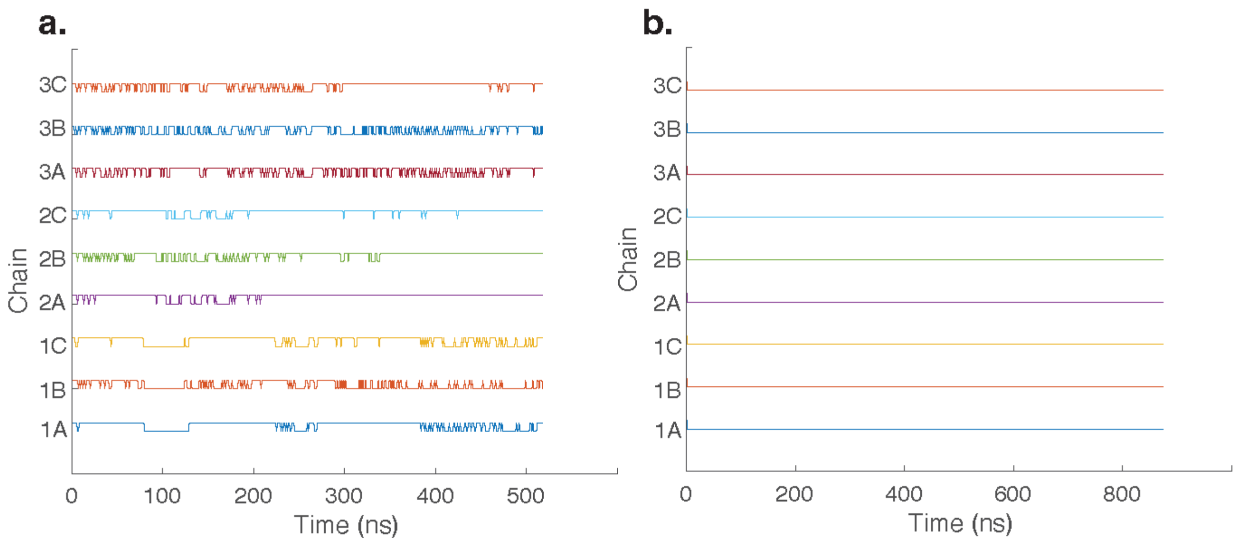

Supplementary Figure S36. Simulations show features of the C1q triple-helical bundle required for non-twisting helices. Plotted are a single triple-helix from the C1q stem region (panel a) and a canonical Pro-Hyp-Gly triple helix (panel b). Each plot displays time courses for nine helical chains (3simulation replicas, 3 chains per replica) scored at nano-second intervals as twisting (0) or non-twisting (1). Scoring was performed at nanosecond intervals by aligning the entire triple helix against a twisting or non-twisting triple helical template and computing the C-alpha RMSD on a per-chain basis. The chain was assigned to the template with the lower per-chain RMSD at that time. The C1q triple helix in panel (a) shows 71% non-twisting content (per-chain range 41-89%), whereas the canonical triple helix in panel (b) shows 0.1% non-twisting content (range 0.1-0.1%). These results support the hypothesis that structural features of the C1q triple helix stabilize a non-twisting conformation relative to a canonical Pro-Hyp-Gly repeat but that inter-helical interactions in the C1q bundle further enhance stability and lock the non-twisting conformation into place.

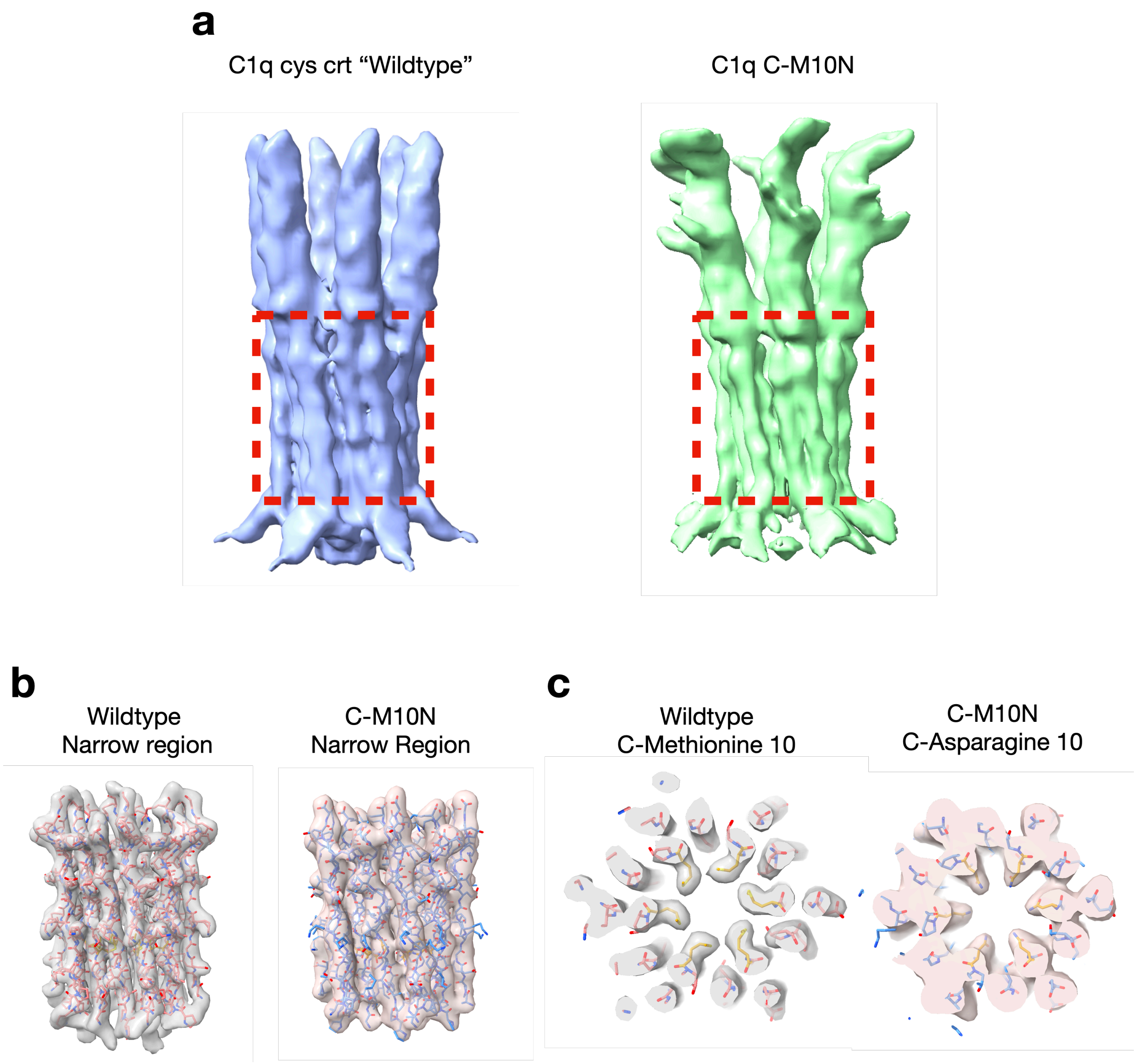

Supplementary Figure S37. Comparison of the C1q cys crt (wildtype) and C1q C-M10 cryo-EM structures. a) On the left the low resolution full C1q cys crt density map is shown. On the right the C1q C-M10N density map is shown. b) Comparison of the side views of the cryoEM structures and models of the narrow region for the wildtype (left) and C-M10N (right) assemblies. c) Top-view comparison of the cryoEM density maps and atomic models for wildtype (left) and C-M10N (right) assemblies in the region corresponding to chain C residue 10.

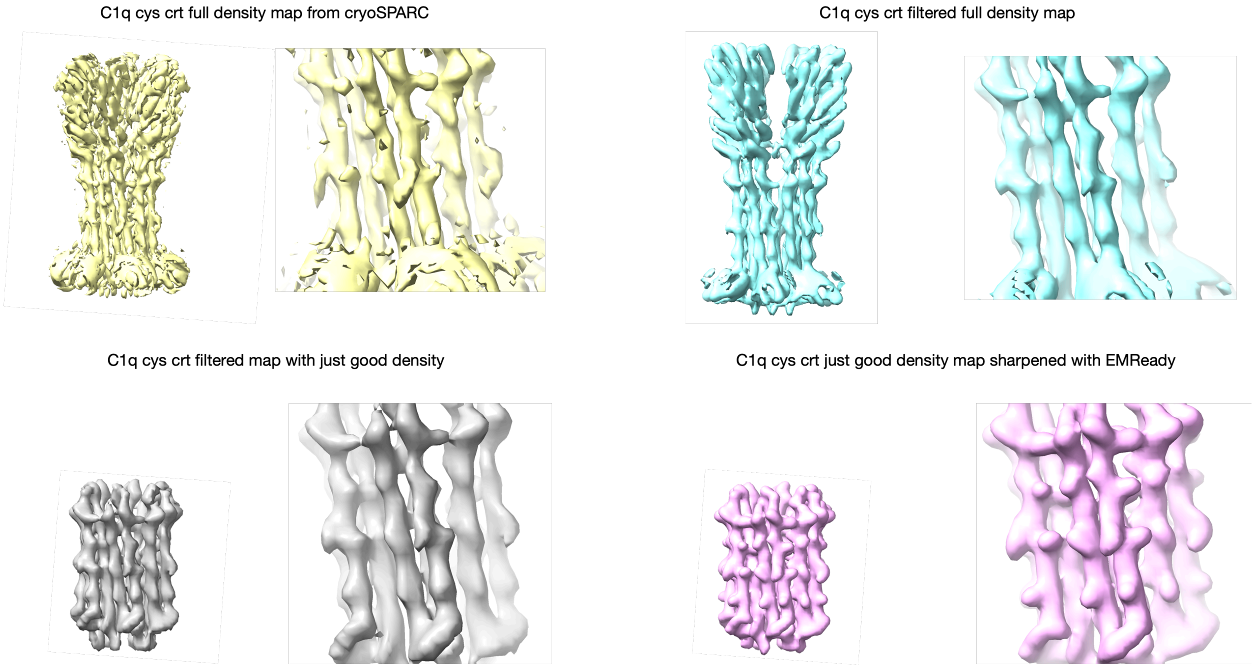

Supplementary Figure S38. Comparison of C1q cys crt density maps after cryoSPARC (top-left), after low-pass filtering the cryoSPARC volume (top-right), after cutting out the good density from the narrow region (bottom-left), and after sharpening the narrow region density map with EMReady (bottom-right). For each map the left image shows the full density map while the right image shows a close-up on side chain features.

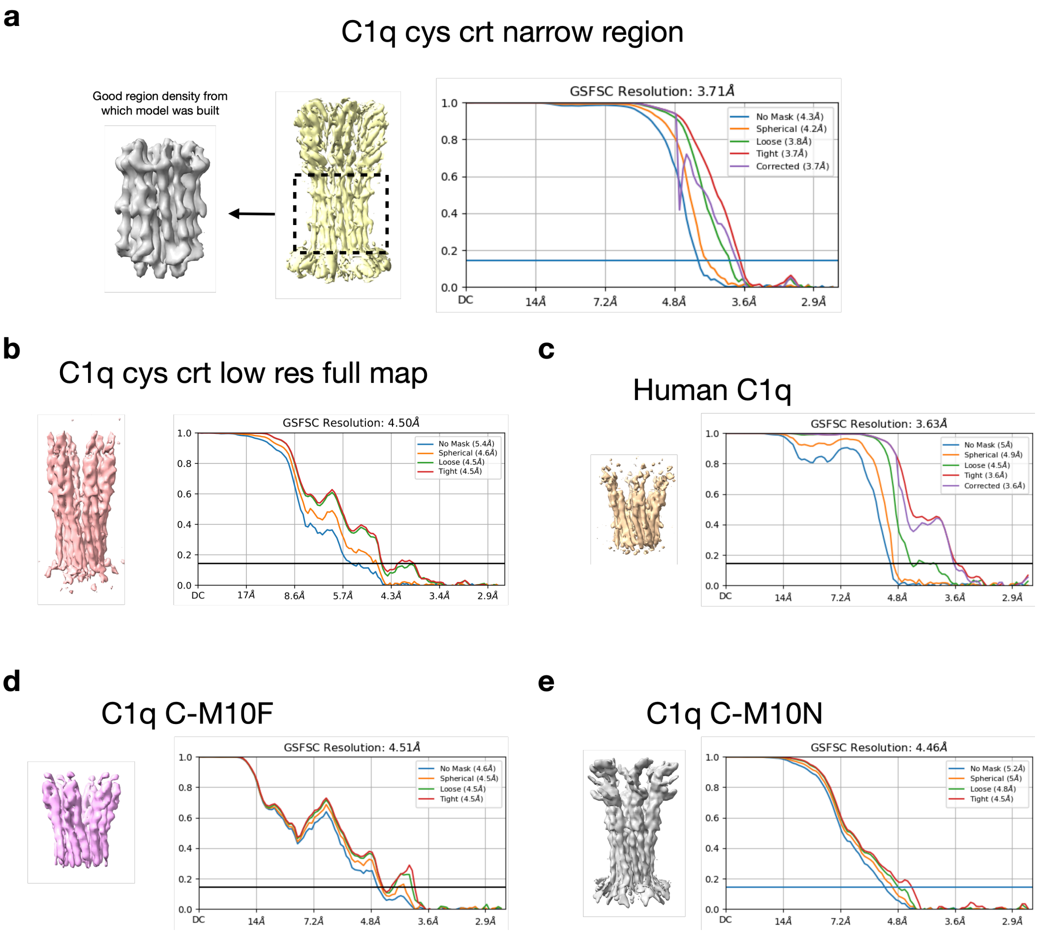

Supplementary Figure S39. Map:Map Fourier shell correlation (FSC) curves for each cryo-EM structure solved in this study. a) Map:Map FSC curve for the reconstruction used to generate the C1q cys crt narrow region map and model. The full density map is shown in yellow feature regions of good density outlined by the black dashed line and shown further in the grey density map. **b)** Map:Map FSC curve for the lower resolution C1q cys crt full density map. **c)** Map:Map FSC curve for the human C1q cryo-EM structure. Note that the 3.6 Å Gold-Standard 0.143 FSC threshold appears to be an over estimate of the resolution. For deposition purposes with this map, we used the 0.5 FSC threshold resolution estimate of 4.5 Å. **d)** Map:Map FSC curve for the C1q C-M10F structure. **e)** Map:Map FSC curves for the C1q C-M10N structure.

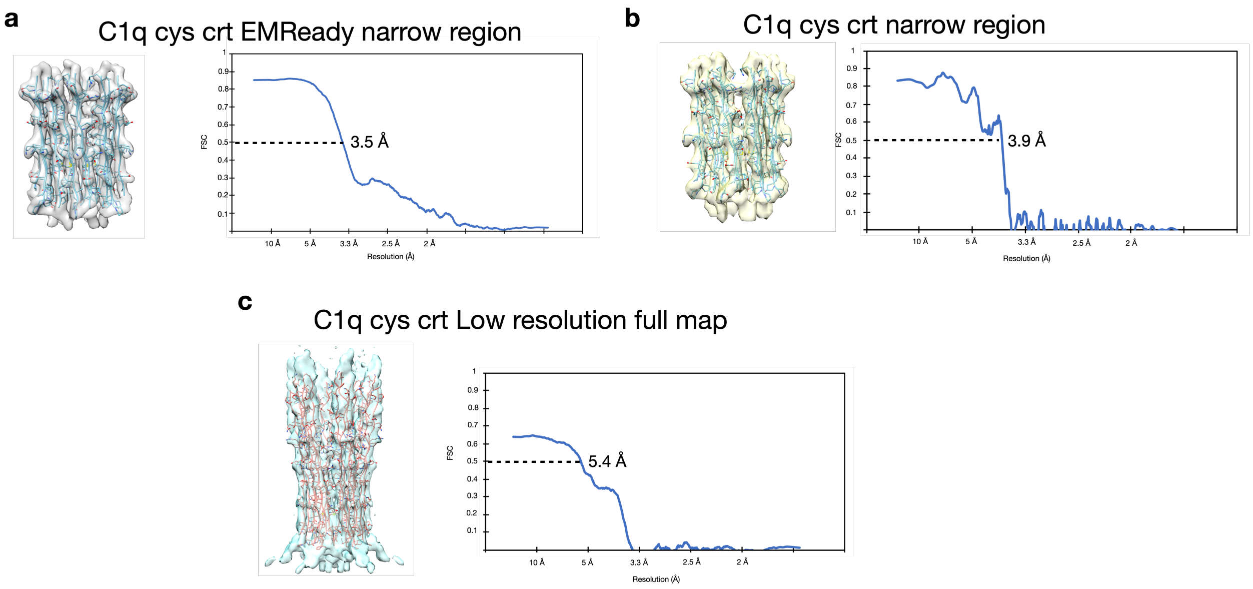

Supplementary Figure S40. Map:Model FSC resolution estimates of the structures with models in this study. The map:model FSC curves are shown for a) the EMReady sharpened density map of the C1q cys crt narrow region, b) the C1q cys crt narrow region map prior to EMReady sharpening, and c) the low resolution C1q cys crt full density map and the extended model.

Additional Files Provided:

Supplementary Movie S1. "C1q_Movie_S1.avi" Provides side views of cryoSPARC 3D variability analysis output.

Supplementary Movie S2. "C1q_Movie_S2.avi" Provides top views of cryoSPARC 3D variability analysis output.
